# Integrating Bacteriocins and Biofilm-Degrading Enzymes to Eliminate *L. monocytogenes* Persistence

**DOI:** 10.3390/ijms26010399

**Published:** 2025-01-05

**Authors:** John A. Renye, Chin-Yi Chen, Amanda Miller, Joe Lee, Adam Oest, Kevin J. Lynn, Samantha M. Felton, Manita Guragain, Peggy M. Tomasula, Bryan W. Berger, Joseph Capobianco

**Affiliations:** 1 Dairy and Functional Foods Research Unit, Agricultural Research Service, United States Department of Agriculture, Wyndmoor, PA 19038, USA; john.renye@usda.gov (J.A.R.J.); amanda.miller@usda.gov (A.M.); adam.oest2@usda.gov (A.O.); peggy.tomasula@usda.gov (P.M.T.); 2 Characterization and Interventions for Foodborne Pathogens, Agricultural Research Service, United States Department of Agriculture, Wyndmoor, PA 19038, USA; chin-yi.chen@usda.gov (C.-Y.C.); joe.lee@usda.gov (J.L.); manita.guragain@usda.gov (M.G.); 3 Department of Chemical Engineering, University of Virginia, Charlottesville, VA 22903, USA; qgg9qv@virginia.edu; 4 Department of Biomedical Engineering, University of Virginia, Charlottesville, VA 22903, USA; smf2qp@virginia.edu

**Keywords:** Listeria, biofilm, enzyme, bacteriocin, intervention, food safety

## Abstract

*Listeria monocytogenes* is a Gram-positive bacterium causing listeriosis, a severe infection responsible for significant morbidity and mortality globally. Its persistence on food processing surfaces via biofilm formation presents a major challenge, as conventional sanitizers and antimicrobials exhibit limited efficacy against biofilm-embedded cells. This study investigates a novel approach combining an engineered polysaccharide-degrading enzyme (CAase) with a bacteriocin (thermophilin 110) produced by *Streptococcus thermophilus*. Laboratory assays evaluated the effectiveness of this combination in disrupting biofilms and inactivating *L. monocytogenes* on various surfaces. The results demonstrated that CAase effectively disrupts biofilm structures, while thermophilin 110 significantly reduces bacterial growth and viability. The preliminary trials indicate a dual-action approach offers a potential alternative to conventional treatments, enhancing food safety by effectively controlling *Listeria* biofilms in food processing environments.

## 1. Introduction

*Listeria monocytogenes* is a facultative anaerobic, Gram-positive, non-sporulating, and psychrotrophic bacterium that causes listeriosis, a severe infection leading to approximately 1600 illnesses, 1500 hospitalizations, and 260 deaths annually in the United States [1]. In the European Union, over 2700 reported illness from listeriosis, 330 hospitalizations and 280 deaths were reported as recently as 2022, with major recent outbreaks in Italy, the Netherlands and Germany [2,3,4]. Hence, listeriosis is a global challenge that imposes a significant threat to food safety across various industries including dairy, as well as on farms and in food processing plants [5,6,7]. Because of these significant health risks across the entire food production chain, *L. monocytogenes* is considered ‘zero-tolerance’ for ready-to-eat foods in the US [8]. *L. monocytogenes* establishes itself on food processing surfaces such as conveyor belts, pipes, and storage tanks via biofilm formation, which enables it to persist despite routine sanitation procedures [9,10,11,12]. It is also established that biofilm-embedded *L. monocytogenes* cells change their shape and alter their growth rate within biofilm-embedded clusters, and these changes correlate with increased persistence and antimicrobial resistance [13]. Hence, biofilm as a key mechanism to cause persistent contamination of *L. monocytogenes* in food processing underscores the pressing need for alternative control strategies that not only disrupt biofilm integrity but also ensure the effective killing of dislodged bacteria and clusters within the biofilm.

A number of different *L. monocytogenes* biofilm structures have been observed depending on cell strain and experimental conditions, including adherent mono- and multilayers as well as more complex 3D structures such as ‘knitted chains’ and ‘honeycombs’ [14,15]. The role of EPS in these observed biofilm structures has been of particular interest, as unlike other pathogens such as *P. aeruginosa*, a specific EPS structure and biosynthetic pathway has not been identified. In a prior study of a wide range of foodborne *L. monocytogenes* isolates, extracellular carbohydrates were present in all strains tested, as well as a positive correlation between increased biofilm formation and carbohydrate concentration [16]. Subsequent work identified D-alanylation of lipotechioic acids as a key pathway that is necessary for biofilm formation, and techioic acids (TAs) were extracted from multiple purified biofilm samples as the primary source of carbohydrates [17,18]. A separate study purified an insoluble, cell-bound poly-β-(1,4)-*N*-acetylmannosamine-containing polysaccharide from a mutant able to form cell aggregates, suggesting additional EPS components beyond TAs may also be present in cell aggregates found in biofilms [19]. Moreover, this mannosamine-containing EPS was responsible for increased cell aggregation as well as higher tolerance to chemical disinfectants, sanitizers, and dehydration, implicating it in enhanced persistence of biofilm-forming *L. monocytogenes* [20]

In many cases, efficacy has been demonstrated for bio-based and chemical treatments or combinations thereof, but in all cases is highly dependent on the specific environment, other microbes present and specific *L. monocytogenes* strains tested [21,22,23]. Testing is often performed on planktonic cells disrupted from biofilms formed on a specific surface; as one example, ethanolic essential oil extracts have been shown to be bactericidal or bacteriostatic in in vitro assays against *L. monocytogenes* cells detached from biofilms formed on stainless steel surfaces in dairy processing facilities [24]. Similarly, chemical cleaning agents such as quaternary amines or peracetic acid were effective in inactivating planktonic cells disrupted from biofilms formed on drains [25]. While a number of variables can affect the performance of a given antimicrobial, the presence of fats, proteins, or other organic materials often greatly reduces the effectiveness of such treatments, including essential oils and chemicals [26]. Other studies have demonstrated that environmental microbiota present in food can also have a significant adverse impact on antimicrobial peptide efficacy against pathogens such as *L. monocytogenes* [27]. In our previous studies, we reported that an engineered polysaccharide-degrading enzyme, CAase, inhibits surface attachment and degrades biofilms formed on glass, plastic, and leafy green surfaces for a broad range of pathogenic bacteria, including *Salmonella*, *L. monocytogenes*, and *E. coli* [28]. Subsequently, we demonstrated this enzyme could disrupt biofilms formed by *L. monocytogenes* and enhance the limit of detection for *L. monocytogenes* in ready-to-eat meat products, demonstrating robust activity in high bioburden conditions where other antimicrobial treatments often exhibit reduced activity [29]. Interestingly, we observed changes in cell wall structure for both *E. coli* and *L. monocytogenes* after enzyme treatment at concentrations below that necessary for inhibiting cell growth, suggesting multiple substrates were targeted by CAase that disrupt both cell-surface and extracellular structures [29].

Bacteriocins are ribosomally encoded peptides with narrow or broad-spectrum antimicrobial activity [30]. Bacteriocins produced by “Generally Recognized as Safe” (GRAS) bacteria, such as nisin and pediocin, have been used extensively as alternatives to chemical antimicrobials for the preservation of foods, including dairy and meat products, eggs, and vegetables [31,32]. The use of bacteriocins as components within hurdle technologies for food preservation may reduce the need for chemical interventions which can negatively affect food quality [33,34]. Additionally, their selective antimicrobial activity has led to commercial interest for developing novel applications for bacteriocins, including their use as sanitizers or therapeutics [30]. In these applications, the bacteriocins would be expected to target specific pathogenic or spoilage bacteria while having little effect on the indigenous microbiota. In one example combining multiple hurdle technologies, a combination of the bacteriocin nisin, phage and buffered dry vinegar fermentates collectively were effective in reducing and inhibiting the growth of *L. monocytogenes* in food [33].

*Streptococcus thermophilus* is a food-grade lactic acid bacteria (LAB) used routinely in the production of yogurt and cheese, with some strains shown to possess a bacteriocin-like peptide (*blp*) gene cluster [35,36]. However, many strains do not naturally produce bacteriocins encoded within this cluster due to the improper functioning of the quorum sensing system that regulates gene expression [37]. *S. thermophilus* B59671 is a rare strain in which bacteriocin production naturally occurs, with the resulting peptide reported to have anti-Listerial activity [38,39]. Additionally, thermophilin 110 was reported to prevent the formation of biofilms by *Streptococcus mutans* and *Cutibacterium acnes* [35,40]. Based on these previous results, thermophilin 110 appeared to be an ideal choice to test for synergistic activity with the biofilm disrupting enzyme CAase. Our results demonstrate that CAase retains robust biofilm degrading activity against *L. monocytogenes* in the presence of thermophilin 110.

Based on our previous research, we investigated the use of CAase in combination with a novel bacteriocin that targets *L. monocytogenes* to address the pressing need for alternative control strategies for biofilm-forming *L. monocytogenes*. Specifically, this study aims to evaluate the combined use of the engineered polysaccharide-degrading enzyme CAase and the bacteriocin thermophilin 110. By examining their combined activity in degrading biofilms and inactivating released cells in in vitro assays simulating food-contact conditions, this work highlights a novel approach that can enhance current biofilm control practices in food processing environments. Collectively, this work demonstrates that combining CAase and bacteriocins is a viable strategy to remove biofilm and inactivate pathogens in systems relevant to food-contact surfaces.

## 2. Results

### 2.1. Biofilm Treatment

**Figure 1:**
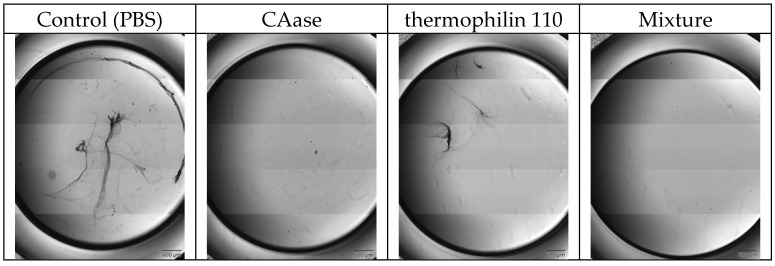
Images of *L. monocytogenes* biofilms following treatment. The images depict mature biofilms following overnight treatment as follows: Control—PBS, CAase—0.1 mg/mL CAase, thermophilin 110–1000 AU/mL thermophilin 110, Mixture—a mixture of 0.1 mg/mL CAase and 1000 AU/mL thermophilin 110.

Figure 1 depicts images captured from the oCelloscope to visually assess the impact of different treatments on mature biofilms. In the control sample, the biofilm appears intact and dense following overnight incubation in PBS. The image demonstrates that the biofilms grown were robust and not significantly damaged by the washing and rinsing processes. The biofilm sample treated with CAase appears to be effectively disrupted and mostly removed relative to the control treatment. This indicates that CAase disrupts the mature *L. monocytogenes* biofilm, as previously reported. Biofilm treated with thermophilin 110 results in an altered biofilm relative to the control, but larger aggregates remain when compared to treatment with CAase. Treatment with a mixture of CAase and thermophilin 110 yields similar results to treatment with CAase alone, demonstrating that the combination of thermophilin 110 and CAase does not enhance or inhibit removal of the biofilm relative to CAase alone.

**Figure 2:**
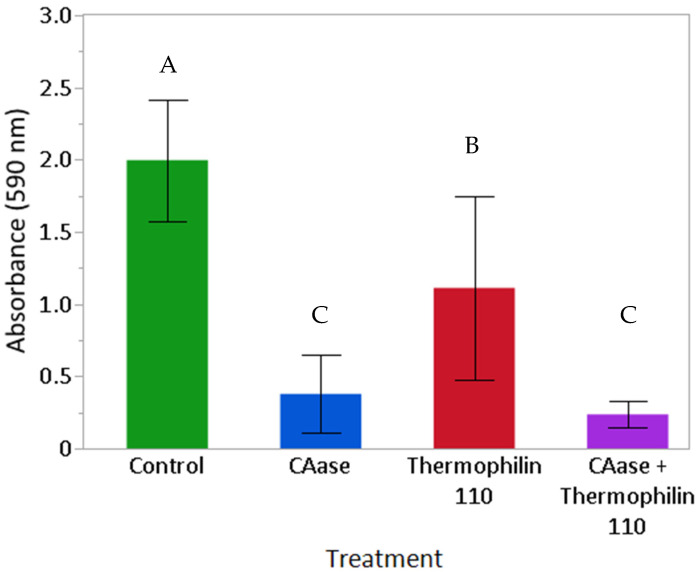
Crystal violet assay results. Biofilm formation on microtiter plates as quantified by absorbance at 590 nm after crystal violet staining as described in Section 4.7. Adsorption at 590 nm is plotted on the *y*-axis and the treatments are presented on the *x*-axis. The bars represent mean absorbance and error bars were constructed using 1 standard deviation from the mean of 3 biological replicates, each with 3 technical replicates. Groups not connected by the same letter were determined to be significantly different (*p* < 0.05) by Student’s *t*-test.

To complement the qualitative assessment of images, crystal violet assays were conducted to semi-quantitatively assess the impact of the treatments on mature *L. monocytogenes* biofilms. Biofilms following PBS, CAase, thermophilin 110, and mixture of CAase and thermophilin 110 treatments (Figure 2) were quantified using a crystal violet staining assay [28,40]. The results are consistent with the images in Figure 1. A reduction in biofilm was seen for all three treatments relative to the PBS control, which were significant (*p* < 0.0001) according to a Student’s *t*-test. When CAase is present, the biofilm appears to be effectively disrupted and mostly removed relative to the control treatment. The average and standard deviation of absorbance measured for CAase with thermophilin 110 were smaller than CAase alone, however the differences between these groups were not significant (*p* = 0.2957) according to a Student’s *t*-test. The results indicate that thermophilin 110 alone could remove more biofilm than the control (*p* < 0.0001), but not as much as when CAase is present (*p* < 0.0001). The response with thermophilin 110 alone displayed variable results as evidenced by the highest standard deviation (0.623) in the group.

### 2.2. Quantifying Inhibitory Effects on Microbial Growth

**Figure 3:**
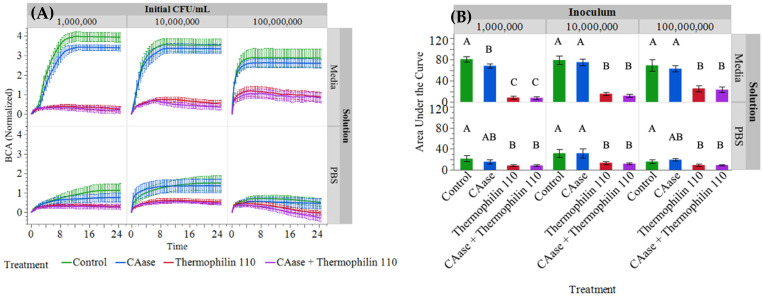
Microscopy image analysis of CAase and thermophilin 110 effects on planktonic Listeria monocytogenes growth. (**A**) The normalized Background Corrected Absorption (BCAN) is plotted on the *y*-axis against time in hours on the *x*-axis. Analyzed images were recorded every 30 min. over the course of 25 h. The points represent an average of 3 experimental replicates, each containing 3 technical replicates (n = 9), and the error bars represent the standard deviation. (**B**) The area under each of the growth curves was calculated using Simpson’s method. The bars represent the average area (n = 9), while the error bars represent the standard deviation. The data were grouped by the composition of the growth media and level of inoculation. Within group comparisons were conducted using Student’s *t*-test and treatments not connected by the same letter were determined to be significantly different (*p* < 0.05).

Time-lapse brightfield microscopy was used to monitor inhibitory effects on microbial growth for 25 h using oCelloScope. The images generated were analyzed using the normalized Background Corrected Absorption (BCAN) algorithm (shown in Figure 3A) and the area under the curve (Figure 3B) was calculated using Simpson’s method.

In media, the growth curves obtained using the oCelloScope for both control and CAase were sigmoidal in nature, indicating bacterial growth for all 3 levels of inoculation. At the lowest inoculum level, there appears to be a reduction in growth when CAase is present relative to the control. In addition to qualitatively assessing the overlap between the error bars, the area under the curves were determined to be different by a Student’s *t*-test (*p* = 0.0204). However, at higher levels of inoculation, the divergence between the growth curves for CAase and the control is less apparent, and there is no difference between their respective areas under the curves (*p* > 0.5516). This indicates that, as the number ratio of CAase to bacteria increases, growth can be impeded which is consistent with previous results [29].

Comparatively, when thermophilin 110 is present in media, there is a significant reduction in *L. monocytogenes* growth for all levels of inoculation relative to the Control and CAase conditions. While there is a degree of sigmoidal character for the growth curves in thermophilin 110 containing media at higher inoculation levels, the curves are significantly flattened, with reduced areas under the curve that differ from the control and CAase conditions (*p* < 0.0001). Thermophilin 110 was evaluated independently and mixed with CAase to assess if there were synergistic effects beyond their individual effects. The results demonstrate that the inhibition of *Listeria* growth in the presence of thermophilin 110 is not enhanced with the addition of CAase. This is shown by the similar shapes of the curves and no significant differences for areas under the curve (*p* > 0.6357). Similarly to what was observed with CAase, the number ratio of thermophilin 110 to bacteria affects the efficacy of the treatment. Specifically, the areas under the curve for *Listeria* growth in the presence of thermophilin 110 alone and in combination with CAase showed a dose dependent response depending on the initial inoculation, specifically between the highest and lowest concentrations (*p* < 0.0104).

Experimental treatments were replicated in PBS buffer to determine if the nutrient composition within LB affected the inhibitory effects of thermophilin 110 with or without CAase. The results are similar to media conditions where both the control and CAase conditions demonstrate growth of the bacteria, though at a significantly reduced rate as evidence by the shape of the curves and reduced area under the curve relative to media (*p* < 0.001). Also, the growth curves in buffer containing thermophilin 110 are significantly flattened but still differ significantly from the control and CAase conditions (*p* < 0.05). Of particular interest is the highest level of inoculation, where the BCAN curves for both control and CAase cultures demonstrate the least growth and the smallest area under the curve. Further, the curves observed in cultures containing thermophilin 110 showed a reduction in absorbance even though there is no significant difference in the calculated areas under the curve for each inoculation used.

**Figure 4:**
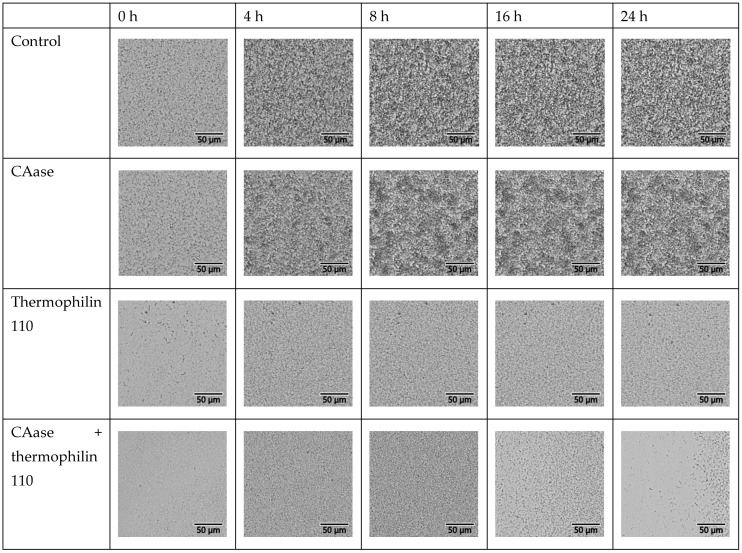
oCelloScope images from media. Images from the oCelloScope that are grouped in rows by treatment and in columns by the time of measurement. The images were collected for the highest level of inoculation.

**Figure 5:**
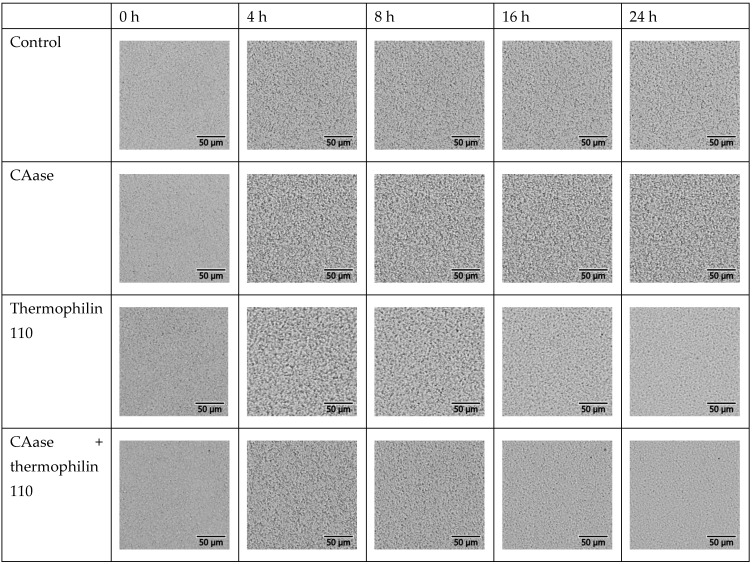
oCelloScope images from PBS. The image displays images from the oCelloScope that are grouped in rows by treatment and in columns by the time of measurement. The images were collected for the highest level of inoculation.

One unique feature of the oCelloScope is the images used to calculate the BCAN can be analyzed to see if they agree with the numerical assessments. Images from the highest level of inoculation in media are presented in Figure 4 while those from the highest level of inoculation in PBS are presented in Figure 5. From visual inspection of the images, the data are largely in agreement with the BCAN measurements. Overall, the density of cells is higher in media than in PBS. Further, over the course of time, the density of cells is significantly lower for both media and PBS when thermophilin 110 is present. Also, there does not appear to be a negative impact on microbial growth when CAase is present. Interestingly, although BCAN measurements did not detect a significant difference between pure thermophilin 110 and its mixture with CAase, visual inspection reveals a lower cell density at 16 and 24 h in the mixture compared to independent thermophilin 110 treatment.

### 2.3. Morphology Assessment

**Figure 6:**
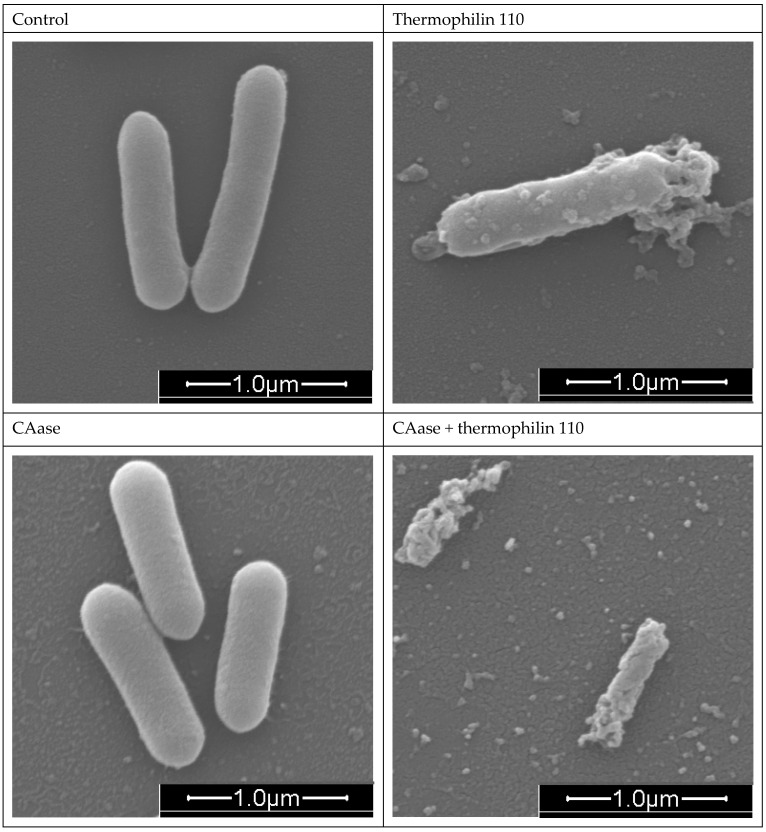
SEM images. *Listeria monocytogenes* cells at 50,000× magnification following 24 h treatment under the following conditions: Control (untreated), Thermophilin 110 (1000 AU/mL), CAase (0.1 mg/mL), and CAase + Thermophilin 110 (0.1 mg/mL CAase and 1000 AU/mL thermophilin 110). The scale bar represents 1.0 µm.

Further examination of cell morphology at the conclusion of the oCelloScope trial (t = 24 h.) involved fixing suspensions on glass slides for SEM imaging, as depicted in Figure 6. Notably, cells under Control and CAase conditions appear as bacilli. In contrast, cells exposed to thermophilin 110 exhibited a distinct, textured surface, indicative of stress. This observation underscores the visual impact of thermophilin 110 on bacterial morphology, further complementing the quantitative data obtained through BCAN analysis.

## 3. Discussion

Emerging treatment modalities based on peptides, proteins, microbes, and other biological approaches offer promise in confronting the challenges posed by *L. monocytogenes* biofilms [21]. With increasing consumer preference for alternatives to chemical treatments, there is greater efforts towards developing bio-based replacements to chemical antimicrobials. Bacteriocins which are naturally produced by food-grade lactic acid bacteria (LAB) are typically defined as low molecular weight peptides which can display narrow or broad-spectrum antimicrobial activities [30]. Many LAB bacteriocins have little or no activity against Gram-negative bacteria, yeast, or molds, but their broad-spectrum activity against Gram-positive foodborne pathogens, specifically *Listeria monocytogenes*, has driven their use as bio-preservatives within the food industry [39,41,42]. Nisin, a bacteriocin naturally produced by strains of *Lactococcus lactis*, has been approved by both the U.S. Food and Drug Administration and the European Food Safety Authority for use as a food additive in a purified form, whereas other bacteriocins have been applied in the form of fermentates [43]. Prior work has described the efficacy of nisin A and a variant M21V (nisin V) against *L. monocytogenes*; in these studies, 10–50 mM nisin A was effective in vitro in inhibiting *L. monocytogenes* growth whereas the modified form, nisin V, was effective at 5–25 μg/mL [44]. Other in vivo studies using purified piscicolin 126 and pediocin PA-1 also demonstrated efficacy in terms of reducing the bacterial load in mouse models of *L. monocytogenes* infection, suggesting their potential to serve as prophylactic treatments for *Listeria* [45,46]. However, in these studies, no evaluation of biofilm-specific effects was described, nor were effects on cell wall structure. A recombinant form of a Lactobacillus-derived bacteriocin (BMP32r) produced in *E. coli* has been described for reducing *Listeria* exopolysaccharide production and killed planktonic *Listeria* cells [47]. Similarly to BMP32r, we observed significant reduction in planktonic cell growth when treated with thermophilin 110 alone (Figure 3); direct imaging of treated cultures confirms that this was due to the lysis of *Listeria* cells, similar to what was shown to occur when *P. acidilactici* and *C. acnes* were exposed to this bacteriocin [35]. Additionally, thermophilin 110 partially reduces *Listeria* biofilm mass (Figure 2), which may be attributed to its antimicrobial activity rather than a direct effect on adhesion (Figure 1). In contrast, treatments involving CAase, either alone or in combination with thermophilin 110, result in the removal of darker extracellular matrix structures, suggesting enzymatic degradation of the biofilm matrix. This degradation likely enhances the detachment of biofilm-embedded cells, contributing to further biofilm reduction. Prior work using thermophilin 110 has demonstrated its effectiveness not only in media and buffer, but directly in milk, whey, and other dairy products at refrigeration temperatures, particularly in combination with pediocin [39]. In this study, direct imaging of samples treated with thermophilin 110 show a distinct, wrinkled and distorted cell structure as compared to untreated cells (Figure 6); in the case of BMP32r, cells remain largely smooth, intact and clustered, suggesting a different mechanism of action for thermophilin 110 versus other bacteriocins such as BMP32r [47].

In food processing environments, persistent *L. monocytogenes* isolates are observed to produce thicker, multicellular biofilms versus environmental isolates, which typically do not aggregate into multicellular clusters and only sporadically adhere to food-contact surfaces [48]. Conventional chemical disinfectants, sanitizers, and antimicrobials used to remove *L. monocytogenes* exhibit limited efficacy against cells within a biofilm [49,50]. Efficacy is further reduced in food and on high bioburden surfaces versus stainless steel or glass surfaces [51,52]. This reduction in efficacy is partly attributed to the limited antimicrobial penetration of the biofilm matrix, which comprises extracellular polymeric substance (EPS), extracellular DNA (eDNA) and protein [53]. Moreover, the adaptability of biofilm-embedded *L. monocytogenes* cells exacerbates their resistance against routine disinfectant and antimicrobial treatments employed in dairy and food processing facilities. The 3-dimensional architecture of *L. monocytogenes* biofilms contains heterogeneously clustered layers of closely packed cells adjacent to regions that are enriched in dead cells and eDNA, shielding cell clusters from antimicrobials [14]. Therefore, an interesting extension of the prior work on thermophilin 110 and other bacteriocins is the effect observed when combined with the biofilm-degrading enzyme CAase. We previously described the use of CAase in reducing biofilm formation and the adhesion of multiple foodborne pathogens on inert surfaces as well as leafy greens [28]. In a subsequent study, we found that the addition of CAase to cultures of *L. monocytogenes* eliminated biofilm formation at lower concentrations below 1 g/L without affecting cell growth and distorted cell surface features and cell shape [29]. Consistent with prior observations, the current study showed substantially reduced biofilm formation, as well as synergism with thermophilin 110 (Figure 2) to essentially eliminate biofilm exopolysaccharide measured using crystal violet staining. As with our prior work, CAase treatment also greatly reduces the adhesion of biofilm-forming cells (Figure 1). Interestingly, in buffer conditions, CAase alone can inhibit *Listeria* growth, depending on the enzyme-to-inoculation cell concentration ratio (Figure 3), though overall the effect of CAase on cell growth is small as compared to thermophilin 110. Our prior work also indicated that, at higher concentrations approaching 1 g/L, CAase could partially inhibit *Listeria* growth [29]. However, in combination, CAase and thermophilin 110 essentially eliminated adherent *Listeria* (Figure 1), and this elimination is consistent with essentially complete removal of exopolysaccharide from solution (Figure 2). While added CAase does not substantially alter cell growth beyond the effect of added thermophilin 110 (Figure 3), direct imaging of cells treated with CAase and thermophilin 110 are noticeably degraded to a larger extent as compared to only thermophilin 110-treated cells (Figure 6). The loss of cell-wall integrity and cell shape is similar to our prior work imaging *E. coli* cells after CAase treatment, in which the cell wall collapsed [28]. Though not definitive, the results of the treatment with thermophilin 110 with CAase are more similar to the results of prior studies using the aromatic terpene linalool as an antimicrobial in terms of changes in cell wall structure [54]. Transcriptomics analysis of *Listeria* treated with linalool revealed several peptidoglycan-associated genes differentially upregulated, again suggestive of a direct effect on cell wall structure. Linalool was able to partially inhibit *Listeria* biofilm, but not completely eliminate biofilm or adherent cells; thus, future work may consider combining CAase, thermophilin 110, and naturally occurring terpenes, such as linalool, which may be advantageous in disrupting multiple aspects of cell wall structure and removing biofilm to fully eliminate persistent *Listeria*.

While this study demonstrates the efficacy of CAase and thermophilin 110 in disrupting *Listeria monocytogenes* biofilms and inhibiting planktonic growth, several limitations should be acknowledged. The experiments were conducted under controlled laboratory conditions, which may not fully replicate the complex environments found in food processing facilities, where variations in temperature, surface materials, and organic load can impact treatment efficacy. Additionally, the study primarily focuses on one strain of *L. monocytogenes* (ATCC 19111), and the results may vary with different strains or serotypes exhibiting distinct biofilm-forming capacities. Although the combination of CAase and thermophilin 110 showed promise, potential long-term impacts on microbial communities and biofilm regrowth were not assessed. Future studies should explore the scalability of this approach in pilot-scale or industrial settings, with a particular focus on environments that simulate food industry conditions. Testing the capacity of CAase and thermophilin 110 to limit *Listeria* biofilm formation in the presence of organic residues, variable surface materials, and environmental stresses will provide valuable insights into their practical application. Additionally, assessing the synergy between CAase and bacteriocins in the context of competing microbial populations will be crucial for understanding their full potential in complex food processing environments.

## 4. Materials and Methods

### 4.1. Reagents and General Procedures

Nanopure water was obtained from a Barnstead ultrapure water purification system (Thermo Fisher Scientific, Inc., Canoga Park, CA, USA). Phosphate-buffered saline (1× PBS) was prepared by dissolving 1 tablet (PBS, Sigma Aldrich, St. Louis, MO, USA) in 200 mL of sterile nanopure water and then filtered with a 0.2 μm sterile filter (Corning, Oneonta, NY, USA). Luria–Bertani (LB) broth (Becton Dickinson, Sparks, MD, USA) was prepared by dissolving 25 g in 1 L of nanopure water and autoclaved according to the manufacturer’s recommendations; 1.5% technical agar (Becton Dickinson) was added to the broth solutions to prepare plates. Crystal violet solution (Millipore Sigma, Burlington, MA, USA) (1% *w*/*v*) was prepared with nanopure water. Piranha solution was prepared by mixing a 3:1 volume ratio of sulfuric acid (Sigma Aldrich, St. Lous, MO, USA) and 30% hydrogen peroxide (Sigma Aldrich).

### 4.2. Bacterial Culture

Isolated colonies of *Listeria monocytogenes* ATCC 19111 grown on LB agar were used to inoculate LB broth. This *L. monocytogenes* strain was originally isolated from poultry and chosen because of its ability to produce a robust biofilm that remains intact even after repeated wash/rinse cycles. Cultures were aerobically grown overnight at 35 °C with shaking at 200 rpm (Innova 42, New Brunswick, Enfield, CT, USA). The overnight culture was adjusted to an OD_600_ of 1.0 (~10^8^ CFU/mL) and serial dilutions were subsequently prepared using LB broth. *Streptococcus themophilus* B59671, which naturally produces thermophilin 110, and *S. thermophilus* ST113 were regularly passaged in tryptone-yeast extract-lactose (TYL) broth, pH 6.5 and grown in static cultures at 37 °C [55].

### 4.3. Biofilm Preparation

*L. monocytogenes* cultures with an OD of 1.0 were diluted to ~10^5^ CFU/mL (1:1000) using LB broth, and 100 μL was added to columns 4–9 and rows C-F of a 96 well plate (TPP, Techno Plastic Products AG, Trasadingen, Switzerland). The remaining outer ring of wells were filled with 200 μL of sterile nanopure water before placing the sealed plate within an atmosphere control vessel, a GasPak EZ CampyPak container system (Becton Dickinson, Sparks, MD, USA), to attempt to minimize evaporation of the culture media during incubation. Cultures were aerobically grown for 72 h at 30 °C with shaking at 120 rpm (Innova 42, New Brunswick, Enfield, CT, USA)

### 4.4. CAase Preparation

CAase was expressed and purified using previously published methods [28]. The enzyme sequence was derived from a predicted Salmonella phage-encoded hydrolase (NCBI Sequence ID YP_004893855.1), optimized for *E. coli* codons, and truncated at the N- and C-terminus to enhance expression. The truncated protein, containing a hexahistidine purification tag, was expressed in *E. coli* BL21(DE3) from a T7 promoter using the pET-28a(+) plasmid with Isopropyl ß-D-1-thiogalactopyranoside (IPTG) induction. Colonies were selected on kanamycin plates (50 μg/mL) and cultured in LB broth containing kanamycin.

These cultures were incubated overnight at 37 °C with shaking at 230 rpm. The culture was scaled up to 250 mL of fresh LB broth and incubated until the optical density at 600 nm reached between 0.6 and 0.8. Protein expression was then induced by adding IPTG to a final concentration of 1 mM and lowering the incubation temperature to 20 °C. After 18 h., cells were harvested by centrifugation.

Cell pellets from an induced 500 mL culture were resuspended in 40 mL of lysis buffer (100 mM Tris, 500 mM NaCl, 10% glycerol, and 10 mM imidazole) and subjected to sonication using a Qsonica at 15 W for 40 min with alternating cycles of 20 s on and 20 s off. The lysate was then centrifuged and the supernatant was collected, discarding any insoluble material. The enzyme was purified using Ni-NTA affinity chromatography on Profinity resin. The column was prepared by removing ethanol and adding nickel chloride, followed by rinsing with water. The lysate was applied to the column and washed with increasing concentrations of imidazole (150 mM and 250 mM) to elute the protein. Fractions were collected, and protein purity was assessed using SDS-PAGE.

Protein samples were mixed with SDS-PAGE buffer and heated at 90 °C for 10 min to denature them before loading onto an acrylamide gel (4% stacking, 12% separating gel). Electrophoresis was conducted at 110 V for 20 min, followed by 150 V for 50 min. The gel was stained with Coomassie blue for two hours and destained with a methanol/acetic acid/water mixture. Typical yields were 25–50 mL of 0.25 g/L CAase after harvest, lysis, and purification from a 1 L shake flask. Purified protein fractions were dialyzed against PBS (pH 8) using a 10,000 MWCO dialysis membrane and then frozen for storage. Protein concentration was determined by measuring absorbance at 280 nm, using an extinction coefficient of 121,990 M^−1^ cm^−1^ calculated from the primary amino acid sequence.

Prior to use, the CAase was centrifuged at 15,000× *g* for 10 min to remove large sized particulates that could potentially interfere with microscopy-based assessments.

### 4.5. Bacteriocin Preparation

Partial purification of thermophilin 110 from cell-free supernatant (CFS) was carried out using a previously described chloroform extraction method [38]. Briefly, CFS was collected from an overnight culture (1 L) of *S. thermophilus* B59671 cultured in TYL broth by centrifugation, mixed with 0.5 volume (500 mL) of chloroform, and stirred vigorously for 45 min. Following centrifugation (13,840× *g*, 4 °C), both the aqueous and solvent phases were removed and discarded. Trace amounts of solvent were removed by running a stream of hot air over the tube. The remaining sediment, which contained thermophilin 110, was dispersed in 10 mL of water. To determine the concentration of thermophilin 110 within the stock suspension, serial two-fold dilutions were prepared and used in an agar well-diffusion assay. Briefly, 50 µL of each dilution were loaded into precast wells in TYL agar seeded with *S. thermophilus* ST113 (1% *v*/*v*), a strain previously shown to be sensitive to thermophilin 110 and used routinely to determine thermophilin 110 concentrations [40]. Thermophilin 110 concentration was reported as arbitrary units (AU)/mL and calculated as the reciprocal of the highest dilution showing a zone of inhibition against ST113 and multiplied by 20. The thermophilin 110 preparation (5120 AU/mL) was stored at −20 °C in 500 µL aliquots. Prior to use, the thermophilin 110 stock was thawed, centrifuged at 15,000× *g* for 10 min to remove particulates, and diluted in sterile PBS or LB media, depending on the experiment, to the desired concentration for treatment. Thermophilin 110 concentration within the recovered supernatant was the same as initially measured in the stock preparation, suggesting the bacteriocin is not associated with the particulates present.

### 4.6. Biofilm Treatment Methods

Following three days of biofilm formation by *L. monocytogenes*, culture supernatant was removed from each well. The wells were gently rinsed three times using 200 μL of PBS. Biofilms were exposed to 150 μL of the following treatments at room temperature for 18 h.: Treatment 1, PBS control; Treatment 2, 0.1 mg/mL CAase enzyme; Treatment 3, 1000 AU/mL thermophilin 110; and treatment 4, mixture of 0.1 mg/mL CAase and 1000 AU/mL thermophilin 110. The treated biofilms were washed three times with 200 μL of PBS. Images of the washed biofilms were captured using a BioTek Lionheart FX automated microscope (Santa Clara, CA, USA) at 60× magnification.

### 4.7. Crystal Violet

The treated biofilms were stained with 200 μL of a 0.1% (*w*/*v*) solution of crystal violet (CV) for 30 min at room temperature. Excess CV solution was removed and washed three times with PBS. Biofilm-associated CV was extracted with 200 μL of 95% ethanol by reflux mixing at room temperature for 30 min. The extracted CV in each well was quantified by measuring absorbance at 590 nm using a BioTek Cytation5 plate reader. Net absorbance was calculated by subtracting background absorbance, which was defined as the average absorbance of wells containing nanopure water which were used to minimize evaporation.

### 4.8. oCelloscope Microplate Assay

Overnight cultures of *L. monocytogenes* in LB were diluted to an OD_600_ of approximately 1.0 (on average 10^8^ CFU/mL). Two 1 mL volumes were centrifuged at 15,000× *g* for 10 min. The supernatant was removed, replaced with 1 mL of sterile PBS, and mixed by vortexing; this process was repeated 3 times. The process was repeated two additional times before combining the two samples. The suspension was serially diluted to prepare 10× and 100× dilutions using 500 μL transfers into 4.5 mL of sterile PBS. Columns of a 96-well plate were charged with 10 μL of 1×, 10×, or 100× dilutions of the *L. monocytogenes* suspension in PBS in triplicate, where each row served as a unique 90 μL treatment. Each treatment was prepared either in LB broth or PBS. Treatment 1 was added to rows A and B, treatment 2 was added to rows C and D, treatment 3 was added to rows E and F, and treatment 4 was added to rows G and H. Rows A, C, and E received treatments prepared in PBS, whereas rows B, D, and F received treatments prepared in LB broth. The 96-well plate was loaded into the oCelloScope and incubated at 30 °C for 25 h. The oCelloscope UniExplorer (v. 12.0) was used to acquire 10 images/well every 30 min using growth kinetics module with BCA (Background Corrected Absorption) analysis algorithm. BCA values as reported from the OCS UniExplorer software V12 as a function of time were tabulated in JMP and graphs were generated. Note that thermophilin 110 and CAase solutions can occasionally contain particulate matter which interferes with oCelloScope images. As a result, these samples were centrifuged at 15,000× *g* for 10 min. to minimize the probability of including debris in the samples.

### 4.9. 6 × 6 Drop Plate

Overnight cultures were 10-fold serially diluted in LB broth. The 6 10-fold dilutions were drop-plated as described previously [56]. Briefly, 12 drops of 7 µL of each dilution were spot inoculated on LB agar plates followed by incubation at 35 °C, inverted, for ~15–18 h. After the incubation period, colonies were counted under a dissecting scope (Zeiss Stemi DV4 Stereo Microscope, Carl Zeiss, Goettingen, Germany) and average colony forming unit (CFU) per milliliter was calculated.

### 4.10. Electron Microscopy

Circular glass coverslips with diameter of 12 mm (Electron Microscopy Sciences, Hatfield, PA, USA) were cleaned with a 3:1 piranha solution for 30 min. The slides were rinsed twice with 50 mL of sterile nanopure water, dried under aseptic conditions, and stored under nitrogen prior to use.

Following 25 h of treatment in the oCelloscope, 80 μL from three of the treatment wells containing the same bacteria concentration were deposited on individual glass slides for SEM imaging. The slides were permitted to statically incubate for 1 h before being fixed in 2.5% glutaraldehyde and prepared for SEM imaging using previously described methods [30]. All SEM images were collected using a Quanta 200 FEG scanning electron microscope (FEI, Hillsboro, OR, USA).

### 4.11. Data Analysis

All the quantitative data were analyzed and graphically presented using JMP software version 14.3.0.

## 5. Conclusions

Overall, our work presents a novel, combinatorial approach to eliminating biofilm-forming *L. monocytogenes* that could have relevance to food processing environments. Biofilm is a major challenge that contributes to persistence in food processing, and *Listeria* in particular exhibits greater antimicrobial and sanitizer resistance in biofilm [12,50,57]. As an enzyme, CAase is continuously active, and therefore can catalyze the breakdown of biofilm exopolysaccharide, exposing cells to antimicrobials to kill *L. monocytogenes*. Thermophilin 110 in particular is an attractive antimicrobial due to its demonstrated effectiveness at refrigeration temperature in dairy products [39]. Additionally, as shown in this study, thermophilin 110 eliminates *Listeria* once released from biofilms and no longer adherent to a surface as well as induces substantial cell-wall damage. With an increasing preference for safer, natural alternatives for control of *Listeria* growth and biofilm persistence, our work provides one possible approach, which can be modified to incorporate other antimicrobials such as linalool that exhibit potentially different modes of action. Future studies will be aimed towards efficacy of CAase and thermophilin 110 as a function of pH, salt concentration, and temperature to further evaluate process conditions, as well as further investigate the underlying mechanism of the cell wall degradation observed, to understand the molecular basis for CAase and thermophilin 110 efficacy.

## Data Availability

Data is contained within the article.
